# The Importance of Nursery Schools

**Published:** 1921-02-26

**Authors:** 


### THE PUBLIC WELL-BEING.

### THE IMPORTANCE OF NURSERY SCHOOLS.

It is rather surprising to find that the *Municipal Journal*, which usually takes a progressive view of matters which affect the public well-being, is purring with satisfaction over the decision of the London County Council to modify very considerably—in reality to postpone—its scheme for the establishment of nursery schools. "The cost of nursery schools," declares this journal in an editorial note, "is undeniably excessive, and many people competent to express an opinion question the value of such institutions, though some people make insidious claims for them." It would be interesting to discover the identity of these very competent authorities, and indeed to find any person intelligently interested in children's welfare who will agree that money so expended "could be spent to better advantage." Even the one instance cited in the newspaper criticism is inaccurate; while noting with severity the fact that "some local authorities" have been so reckless as to establish day nurseries, the journal asserts that a report prepared by the Bournemouth Medical Officer of Health shows that it costs £40 or £45 a year to look after a child for five days of the week; but it neglects to add that in this case the mother pays at least £15 of the cost, so that the ratepayers do not contribute more than about £25. "Poor, indeed, must be the district," we are informed, "which has not sufficient kindly disposed ladies willing to co-operate to mind the babies of the small proportion of mothers who have 'to put their babies out.'" It is quite evident that the writer, if he has ever heard of the East End of London has never been there, and that he is not familiar with observations made on the subject by the Chief Medical Officer of the Board of Education.

These observations have been scarcely mentioned in the daily press, constituting as they do a small section of a comprehensive report recently issued by the Chief Medical Officer on the work of the schools in relation to health; but they are important because they indicate the true character of the nursery-school experiments, about which public ignorance is general. The powers given to local education authorities for supplying or aiding the supply of nursery schools relate only to children "whose attendance at such a school is necessary or desirable for their health and physical or mental development"; and authorities are, moreover, expressly empowered to attend "to the health, nourishment, and physical welfare of children attending nursery schools." These schools are, therefore, concerned mainly with children living in the poorer districts of large towns and where housing conditions are bad, overcrowding common, and infant mortality high—an environment, in short, from which many factors of healthy nutrition are certainly absent, and where the resistance of the body to the onset of disease is not likely to be strengthened. The primary duty of the nursery school is nurture, the care of the health of the children, and the prevention of future disease. Children between two and five years have passed the perilous period of infancy, but they need continued attention to nutrition and general hygiene if they are to grow into strong and healthy children. They are highly susceptible to various childish ailments, notably to infection and respiratory diseases, at this early age particularly dangerous.

Hence the importance of small schools and much individual care of the children. Such schools should be closely related to elementary schools, day nurseries, and infant welfare centres. The *Municipal Journal* appears to have confused day nurseries with nursery schools and not to know very much about either. We hope that, as soon as the present financial stress is eased, the London County Council and other authorities in the kingdom will continue the development of these schools. Four training colleges which conduct courses of training for nursery-school teachers have either provided nursery schools themselves or else have nursery schools closely connected with them which they use as demonstration schools for the training of their students. The Rachael McMillan Nursery School is also used in connection with the training of the students attending the courses for certificated teachers which are conducted at Deptford. Some twenty-five or thirty other proposals for nursery schools are now under the consideration of the Board of Education. In some of these latter cases the schools are already in existence and are doing good work, and it is understood that the Board are only awaiting information in regard to certain details before granting them formal recognition. Apart from the proposals which have actually been submitted, many local education authorities are known to be contemplating the provision of a number of experimental nursery schools in suitable districts in their areas, and it is hoped that with whatever assistance may be available from the rates and from the Exchequer further voluntary efforts in this direction will soon be forthcoming. The total grant paid by the Board during the past financial year in respect of the provision and maintenance of nursery schools was only £2,262, so that it is evident that they do not at present represent more than a quite insignificant item in educational expenditure.

